# The Use of Convection-Enhanced Delivery with Liposomal Toxins in Neurooncology

**DOI:** 10.3390/toxins3040369

**Published:** 2011-03-31

**Authors:** Massimo S. Fiandaca, Mitchel S. Berger, Krystof S. Bankiewicz

**Affiliations:** Molecular Therapeutics Laboratory, Department of Neurological Surgery, University of California San Francisco, 1855 Folsom Street, Mission Center Building, Rm. 226, San Francisco, CA 94103, USA; Email: Bergerm@neurosurg.ucsf.edu (M.S.B.); Krystof.Bankiewicz@ucsf.edu (K.S.B.)

**Keywords:** blood-brain-barrier, convection-enhanced delivery, diffusion, glioma, liposome, neurooncology

## Abstract

Liposomes have long been effective delivery vehicles for transport of toxins to peripheral cancers. The combination of convection-enhanced delivery (CED) with liposomal toxins was originally proposed to circumvent the limited delivery of intravascular liposomes to the central nervous system (CNS) due to the blood-brain-barrier (BBB). CED offers markedly improved distribution of infused therapeutics within the CNS compared to direct injection or via drug eluting polymers, both of which depend on diffusion for parenchymal distribution. This review examines the basis for improved delivery of liposomal toxins via CED within the CNS, and discusses preclinical and clinical experience with these therapeutic techniques. How CED and liposomal technologies may influence future neurooncologic treatments are also considered.

## 1. Introduction

The effective delivery of therapeutic agents via the vasculature to the central nervous system (CNS) is significantly affected by the presence of the blood-brain-barrier (BBB) [1]. The endothelial cells of the BBB differ from those in other vascular locations, since they lack fenestrations and have more extensive tight junctions (TJs), while lacking significant pinocytic vesicular transport [2]. In addition, the BBB features an acellular basement membrane immediately beneath the endothelial cells, and two other cellular components, the pericytes and astrocytes encompassing and completing this limiting structure to ingress into the CNS [2]. While the TJs significantly reduce the paracellular passage of hydrophilic molecules into the brain [3], O_2_, CO_2_, and small lipophilic molecules easily diffuse across the cell membranes, driven by their concentration gradients [4]. Specific membrane transporters are available for the uptake of glucose and amino acids from the blood, while many macromolecules are taken up via receptor-mediated endocytosis [5,6,7]. All of these components, working in concert, are essential to the homeostasis of the CNS provided in part by the BBB.

In neurooncology, the BBB is often disrupted in association with the intrinsic growth of tumors within the CNS parenchyma [8,9]. While some feel that the BBB does not play a significant role in “impeding the success of brain tumor chemotherapy” [10,11,12], most feel that the presence of the BBB significantly reduces the effective delivery of intravascular chemotherapeutic agents to the brain [13]. The major strategies developed to improve chemotherapeutic delivery to brain tumors involve designing drugs or methods with improved permeability to the BBB [14,15,16], delivery strategies that feature BBB disruption [17,18,19], or by circumventing the BBB altogether by intrathecal cerebrospinal fluid (CSF) delivery [20,21,22,23], or intraparenchymal delivery that excludes convection enhanced delivery (CED) methods [24,25,26,27,28,29]. All of these approaches attempt to achieve turmoricidal drug levels and increased contact time within, and in proximity to, the brain tumor, and thereby provide effective treatment [13,30].

Over the last 10–15 years, local drug delivery, bypassing the BBB, has gained momentum by offering expanded capabilities to the magnitude and types of drugs that can be delivered within CNS for the treatment of neurooncologic pathologies [31], and by delivering therapeutic levels of chemotherapeutic agents within brain parenchyma compared to other delivery modalities [30]. The remainder of this review directs attention to two particularly appealing delivery modalities for use with neurooncologic chemotherapeutic agents, CED and liposomes. Although developed independently, these two delivery options have been recently combined in an effort to improve efficacy in the treatment of CNS malignancies. Details of both modalities will be explored and their future prospects in neurooncology considered.

## 2. Diffusion *versus* Convection-Enhanced Delivery (CED)

**Figure 1:**
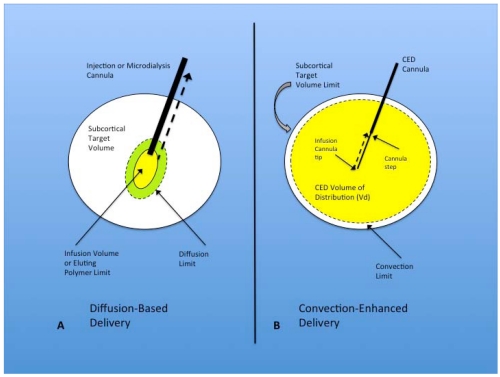
(**A**) Diffusion-based delivery system. A characteristically larger injection cannula is used to deliver the infusion volume within the target region for direct injection and microdialysis. The infusion volume typically displaces the surrounding parenchyma at the tip of the cannula and forms a small cavity from which diffusion occurs into the surrounding brain, eventually expanding to the diffusion limit, but falling far short of filling the subcortical target volume. Implanted polymers filling the infusion volume show similar diffusion volume. Another factor that limits the effectiveness of this technique is the development of backflow or reflux (dashed black arrow) of the infusate out of the target region, along the path of the injection cannula. This is seen most often with larger cannulae; (**B**) Convection-enhanced delivery system. Optimal CED cannulae are narrow (~165 µm) and are attached to the pump mechanism that controls the rate of infusion. The infusion cannula extends for a distance beyond the outer guide cannula, with the transition between the two called the cannula step. The infusate is delivered with a constant flow rate (most commonly 0.2–5.0 µL/min) from the infusion cannula tip. This flow rate establishes a pressurized extracellular bulk flow that allows the homogenous distribution of various sized molecules/particles significant distances from the infusion cannula tip. Reflux (dashed black arrow) typically only occurs up to the cannula step, and major backflow along the cannula and out of the target region prevented by central placement of the step within the target volume. The convection limit can more easily approach the subcortical target volume limit.

To better understand the basic physiology and distribution mechanisms associated with CED, it is important to contrast it with diffusion. Diffusion-based delivery mechanisms are essential to the distribution of chemotherapeutic agents within the brain parenchyma following intravascular delivery, intrathecal cerebrospinal fluid infusions, direct brain injections, elution from implanted polymers, and via microdialysis (Figure 1A). With all of these distribution options, therapeutic agents disperse through the extracellular space (ECS) according to their concentration gradient and inversely proportional to their molecular size [32,33,34]. Chemotherapeutic agent diffusion does not typically extend for greater than a few millimeters from the site of greatest concentration with the modalities listed above [35], and, especially for smaller molecules, can be impacted by capillary clearance and metabolism [36,37,38], affecting the local ECS microenvironment. To date, delivery of chemotherapeutic agents utilizing these diffusion-based technologies are exceedingly difficult to standardize and control [39]. Diffusion, unfortunately, provides a limited and heterogeneous distribution of therapeutics in the normal brain ECS [40], and that associated with gliomas [41,42], due in part to its mechanism of action and intrinsic parenchymal factors [39,40,41,42,43]. 

In contrast to diffusion, CED is a delivery modality within the brain ECS that utilizes bulk flow, or fluid convection, established as a result of a pressure gradient [44], rather than a concentration gradient (Figure 1B). Through the maintenance of a pressure gradient from the delivery cannula tip to the surrounding tissues, CED is able to distribute small and large molecules, including high molecular weight proteins, to clinically significant target volumes [44,45], centimeters rather than millimeters in diameter. Viruses and other large particles [46], including liposomes [47], are also easily distributed within the brain via CED. The advantages of CED over diffusion, therefore, include: (i) expanded volume of distribution (Vd); (ii) a more uniform concentration of the infused therapeutic within the target Vd; (iii) delivery of the vast majority of the infused therapeutic within the target volume [45]. 

**Figure 2:**
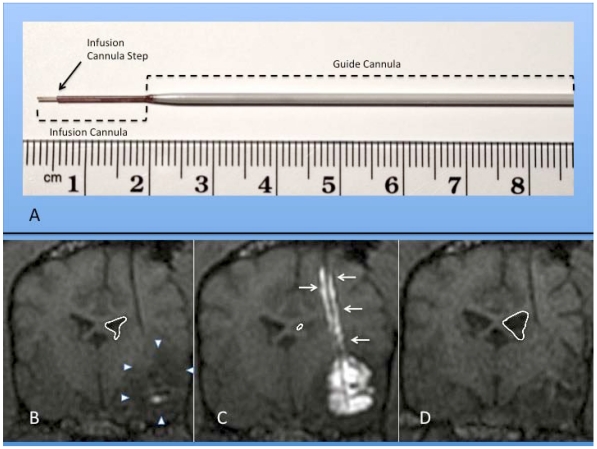
(**A**) Photograph of actual step cannula used for CED. The guide cannula is stereotactically placed within brain tissue to just above the intended target. The delivery cannula is then passed through the guide to reach the final target point. (B-D) Coronal MR images of RCD using mixture of gadolinium liposomes and liposomes carrying CPT-11 in a canine with temporal lobe glioma (outlined by white arrowheads); (**B**) Pre-CED image following catheter placement within tumor mass. Notice ventricular mass effect on ipsilateral ventricle (outlined in white); (**C**) RCD with significant filling of tumor volume with contrast. Note contrast reflux along guide cannula (small white arrows) and increased mass effect on ventricle; (**D**) Coronal MRI one month after CPT-11 RCD. Notice decrease in mass effect on ipsilateral ventricle and temporal lobe in this canine patient.

Our understanding of CED distribution has been amplified by the realization that arterial pulsations within the brain’s perivascular spaces enhances the distribution of convected therapeutics [48], and by a better appreciation of the complexities of the extracellular matrix and its effects on convection [49,50,51], and consideration of the biophysical properties of the ECS volume fraction [43]. Technical CED infusion parameters, such as cannula size and shape (Figure 2), infusion rate (usually 0.2–5.0 µL/min or 0.012–0.3 mL/h), infusate concentration, and tissue sealing time, have been defined and refined to improve distribution of therapeutics [46,52,53,54], while limiting potential toxicities and morbidities [46,54,55]. A major advance in the safe and efficacious use of CED in clinical neurosurgery has been the development of real-time convective delivery (RCD) [56,57,58], which currently utilizes magnetic resonance (MR) imaging to visualize the CED process with the aid of co-convected contrast agents (Figure 2) [55,59,60,61]. The use of RCD has become critical in allowing treating physicians to directly monitor the distribution of therapeutics within the brain. Reflux along the CED catheter or leakage outside the target area, especially at higher flow rates, can be monitored and corrective steps taken, such as retargeting the catheter or altering the rate of infusion [39,62]. 

Several recent human clinical trials that utilized CED for the delivery of therapeutics to the brain without RCD have been regarded as not meeting clinical endpoints, including trials for treatment of neurodegenerative disease [63,64,65,66], and neoplastic conditions [67,68,69,70,71,72,73]. It remains unclear as to whether the inconclusive results in these trials could be: related to lack of efficacy of the therapeutic; due to variability in response of patients to the therapeutic; due to lack of consistent volumetric delivery of the therapeutic to the target; or, to some additional factor(s) yet to be confirmed. Lack of effective monitoring of the infused therapeutics, without imaging, and the likelihood of poor drug distribution in these human trials has led to recurring criticisms [74,75]. 

Similarly, in the first comparative Phase III trial of CED delivered chemotherapy *versus* Gliadel wafer (diffusion-based eluting polymer) therapy for recurrent glioblastoma treatment [75], no significant survival difference was seen between the two groups. Intrinsic tumor barriers and parenchymal effects may be the primary forces influencing the distribution of convected chemotherapeutic agents [76]. Phenotypic characteristics associated with glioblastoma multiforme (GBM) include rapid growth, high glucose consumption, intra-tumoral necrosis, hypoxia, and vasogenic brain edema [77]. Although greater edema should allow facilitated diffusion due to a larger ECS, diffusion appears impeded in tumors due to altered extracellular matrix composition [41]. Tumor malignancy grade strongly corresponds to an increase volume of the ECS accompanied by structural changes manifested by increased barriers to diffusion for small molecules [41]. Whereas in low-grade tumors the diffusion of molecules is reduced mainly by the presence of a dense network of tumor cell processes, the barriers to diffusion within the ECS of high-grade gliomas is caused by the overproduction of certain glycoprotein components of the extracellular matrix (ECM), mainly tenascin [42,78]. ECM glycoproteins not only stabilize the ECS volume, but also serve as a substrate for adhesion and subsequent migration of the tumor cells through the enlarged ECS. These same alterations in ECS structure may hinder the diffusion of certain neuroactive substances or therapeutic molecules within neoplastic tissue [78]. These barriers to diffusion and convection, directly related to the tumor parenchyma, provide a less permeable medium for CED, and even less so for diffusion-based delivery options.

While interstitial fluid velocity measurements *in vivo* are difficult to assess, investigators have developed mathematical models based on physical principles to predict fluid transport that occurs during CED in normal and neoplastic brain tissue [79,80]. These models predict that the tumor core maintains an elevated interstitial fluid pressure [79], and that tumors have an outward flow of extracellular fluid (ECF) at their periphery [80]. For CED to adequately perfuse a neoplasm, therefore, this pressurized outward flow of ECF from the tumor core must be overcome, unless the delivery catheter is somehow centered in the lesion’s center of pressure [76]. Despite adequate coverage of a tumor volume with an effective therapeutic via CED, the rapid clearance of the drug due to this outward flow of ECF (and reduced concentration-time product) may provide little or no clinical efficacy [76,81]. Such interstitial pressures and fluid flows make it even less likely that peripherally placed diffusion-based therapeutics will influence the tumor core. The use of RCD in future neurooncologic and neurodegenerative disease trials may allow better differentiation of efficacy between CED and diffusion-based treatment modalities, but will also allow direct visualization of the Vd of convection therapies, and allow a better estimation and standardization of the therapeutic contact time. 

## 3. Liposomes

**Figure 3:**
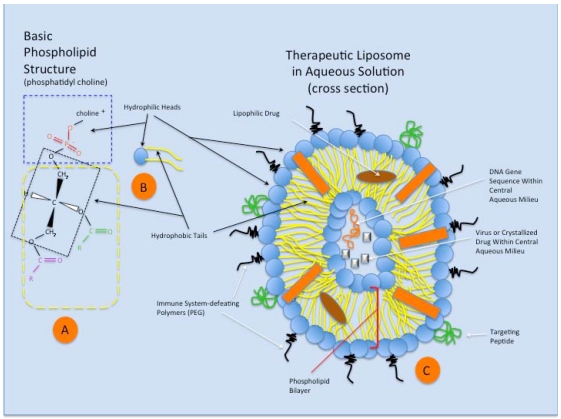
A schematic representation of the phospholipid structure and that of a theoretical therapeutic liposome in aqueous solution. (**A**) Phosphatidyl choline is a typical double chain amphiphile with the steric characteristics that preferentially forms bilayers and liposomes. The hydrophilic head (blue dashed rectangle) of the molecule is charged and contains the anionic phosphate group and that cationic choline molecule, attracting water to its domain. The glycerol molecule (within black dotted rectangle) connects the hydrophilic end of the amphiphile to two fatty acids (typically of different lengths (purple and green groups), which make up the hydrophobic tails (yellow dashed rectangle); (**B**) A schematic representation of the phosphatidyl choline amphiphile (or other double chain amphiphile), featuring the hydrophobic head (blue) and hydrophilic tails (yellow); (**C**) Schematic representation of a theoretical therapeutic liposome in aqueous solution, seen in cross section. The double chain amphiphiles arrange themselves in a spherical bilayer vesicle, with water surrounding the outside of the liposome and retained within the central aqueous milieu. Cholesterol in the membrane (orange rectangle) stabilizes the liposome structure. Complex targeting molecules (green) are shown on the outer surface of the bilayer, allowing preferential binding of the liposome to targeted cell surface receptors for cellular uptake. Immune system-defeating molecules (black) (e.g., polyethylene glycol, PEG), through their enhanced steric effects, increase the ability of the liposome to avoid clearance via phagocytosis. Within the phospholipid bilayer (red brackets), lipophilic drugs can be assimilated and transported via the liposome. Finally, small and large molecular species can exist within the liposome’s aqueous core (based on liposomal size), including proteins, drugs, genetic material, viruses, and other particulates, for eventual incorporation within the target cell.

Liposomes have been included into a group of phospholipid nanoparticles, that form a “core-shell structure” [82,83,84], since their initial description by Bangham [85,86,87], and which can be used to carry various therapeutic agents. Liposomes are typically composed of double chain phospholipid amphiphiles (chemical compounds with combined hydrophilic and lipophilic properties) in combination with cholesterol, forming spheroidal bilayer membrane structures that encompass an aqueous internal domain [83,88] (Figure 3). The length of the fatty acid chains and the presence or absence of double bonds within the bilayer lipids affects the membrane fluidity, as does the combination of different phospholipids within the membrane structure [89,90]. The cholesterol strengthens and stabilizes the bilayer membrane [91], and reduces cation leakage in physiologic systems [92]. Increasing the molar cholesterol content of liposomal drug carriers reduces the release kinetics of the therapeutic [93]. Specific liposomal properties, therefore, can be tailored by the membrane component makeup [94,95], and most recently through the combination of polymer nanoparticle technology with liposomes [96]. 

**Figure 4:**
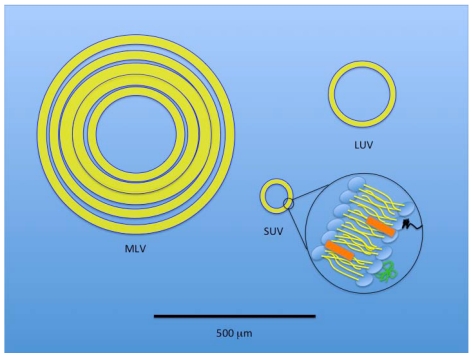
A size-based, schematic representation of the three basic liposomal structures. Black line at bottom of the figure represents 500 µm. A multilamellar vesicle (MLV) has layered membranes separated by minimal aqueous volume and is typically a larger structure that provides an increased hydrophobic volume for better incorporation of lipophilic drugs. A large unilamellar vesicle (LUV) provides increased internal volume for incorporation of hydrophilic therapeutics. A small unilamellar vesicle (SUV) is also depicted with an expanded view of the typical component membrane structure shared by all three liposomal types.

Liposomes are typically formed by the addition of energy to amphipathic phospholipids in aqueous solution. Liposomal structures can range from long tubules to spheres, with dimensions from several hundred Angstroms to fractions of a millimeter [97]. A prototypical liposomal vesicle has a single, closed lipid bilayer confining a single internal aqueous volume. The three basic types of liposomal structures include multilamellar vesicles (MLV, typically > 500 nanometers (nm)), small unilamellar vesicles (SUV, <100 nm), and large unilamellar vesicles (LUV, ≥100 nm) [94,97,98] (Figure 4). Sonication of phospholipids in an aqueous solution can produce liposomes [97], but in extreme circumstances can damage the vesicles. Low shear conditions favor the development of MLVs, while increasing shear produces LUVs, and finally SUVs.

Regardless of the preparation methodologies [99,100,101,102,103,104,105,106,107,108,109,110,111], liposome formation results from the addition of energy (e.g., heating, sonication, homogenization, shaking, *etc.*) altering the tendency for lipid membranes to form a flat bilayer at an aqueous interface, and instead form bilayered vesicles [112]. Unfortunately, only a few of the conventional liposomal production methods are capable of entrapping large quantities of water-soluble agents [107]. Conventional liposomal production methods, such as the reverse-phase evaporation technique [101], ether injection/vaporisation technique [99,100], and freeze-thaw method [102], produce a heterogeneous mixture of large unilamellar vesicles (LUV) or multilamellar vesicles (MLV) [112]. Production of a more homogenous liposome mixture has been accomplished through centrifugation [113], or filtering methods [114,115].

Typically, liposomes within the circulation are quickly coated with opsonizing plasma proteins, taken up by phagocytic cells within the reticuloendothelial system (RES) (see next section, Cellular Uptake of Liposomes), and rapidly cleared from the bloodstream. The addition of polyethylene glycol (PEG) or derivatives to the external membrane surface of liposomes (PEGylation) has proven effective in inhibiting RES clearance and thereby increasing plasma circulation time [116,117,118]. The mechanism of PEGylated liposome longevity has been investigated [119], and postulated to be primarily due to a protective conformational cloud of steric interference on the liposomal surface associated with the flexible hydrophilic polymers. Such a protective surface also alters surface charge characteristics, and reduces opsonization and phagocytic clearance. 

From the circulating bloodstream, liposomes of small and large diameters are able to diffuse across the BBB due to their lipophilic characteristics. SUVs modified with brain transport molecules on their surface can also undergo receptor-mediated or absorptive-mediated transcytosis [98]. Within the brain, liposomes present little or no toxicity to the host [120], and most commonly enter cells within the CNS via endocytosis. Upon entering the CNS ECS, liposomal diffusion is markedly limited due to their size and cellular binding characteristics.

## 4. Cellular Uptake of Liposomes

Liposomes were first shown to be effective intracellular transport vehicles for substances that typically did not gain access to the intracellular space in 1974 [121]. Two major pathways for cellular internalization of liposomes are present: phagocytosis, and endocytosis [122]. Phagocytosis occurs primarily in professional phagocytes (e.g., macrophages, monocytes, neutrophils, and dendritic cells) [123]. Fibroblasts, endothelial cells and epithelial cells have some phagocytic capabilities but to a much lower extent [124]. Opsonization or tagging of the nanoparticles for phagocytosis, is effectively carried out by serum proteins, including immunoglobulins, complement components, laminin, fibronectin, C-reactive protein, and type-I collagen [125,126]. Opsonized particles specifically attach to phagocytes via receptor-ligand interactions, which trigger a signaling cascade that results in actin-dependent pseudopodia extension and eventual engulfing of the particle, ingestion, and processing through phagolysosomes [122]. The entire process can take 30 min to 2 h and is highly dependent on surface properties of the ingested particle [123]. Particle size matters in phagocytosis, with the process optimized for particles greater than 250 nm, and with smaller particles less efficiently internalized [127]. Larger sized liposomes show increased opsonization by serum proteins and phagocytic clearance, a process that has been largely defeated in the peripheral circulation through the use of polyethylene glycol (PEG)-coating on liposomes [119], and other nanodelivery vehicles [122,128]. Additionally, liposomes with a significant surface charge (positive or negative), have a much higher binding affinity to phagocytes than vesicles that are neutral, and hydrophobic nanoparticles are more readily taken up than hydrophilic non-ionic ones [122]. Finally, shape and rigidity of the liposome or nanoparticle also influence the likelihood of cellular uptake via phagocytosis. Less spherical and more rigid particles directly stimulate phagocytic ingestion [122]. 

**Figure 5:**
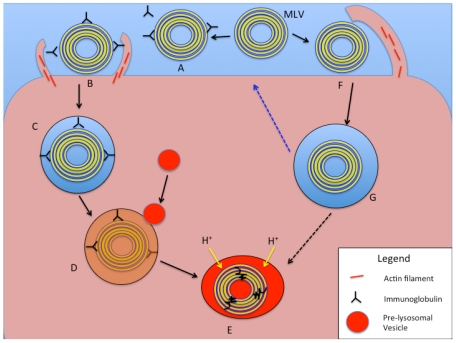
Schematic representation of intracellular processing of large liposomal nanocarriers (>250 nm diameter) based on phagocytosis and macropinocytosis mechanisms. In the phagocytosis pathway (A–E), an MLV (or LUV) is opsonized with serum proteins or immunoglobulins (**A**). Receptor binding on the cell surface to opsonins results in actin assembly and particle engulfment (**B**), leading to the formation of a phagosome (**C**). With maturation of the phagosome, pre-lysosomal vesicles fuse with it and release degradative enzymes (**D**), and finally form a phagolysosome with acidification and degradation of the liposomal vesicle and contents (**E**). In the macropinocytotic internalization pathway (F,G, and E), large membrane protrusions non-specifically engulf a large amount of ECF, including liposomes of various sizes (**F**). The fate of the resulting macropinosome (**G**) includes processing of its contents via acidification and fusion with enzyme-rich vesicles to form a phagolysosome (E). An alternative pathway (blue dotted arrow) for the macropinosome is to fuse with and recycle its content to the cell surface.

**Figure 6:**
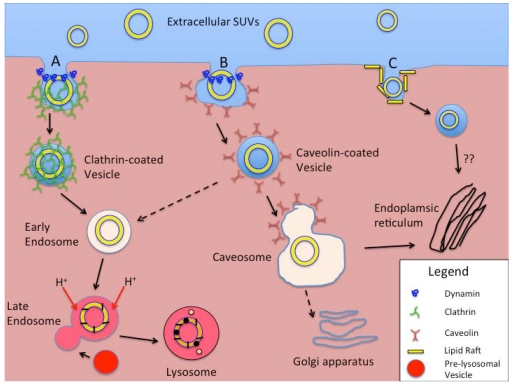
Schematic representation of intracellular processing of smaller liposomal nanocarriers (e.g., SUVs). Extracellular SUVs interact with the cell membrane and are taken up by (**A**) Clathrin-mediated endocytosis (CME), (**B**) Caveolin-mediated endocytosis (CvME), or (**C**) Non-clathrin- or non-caveolin-mediated endocytosis (NCME). With CME (A) clathrin coated pits form either as a receptor-mediated event or spontaneously, the latter process called fluid-phase endocytosis. Vesicle fission from the membrane is mediated by the GTPase dynamin. Coated vesicles are internalized, shed their clathrin coats, and develop into early endosomes. With acidification and fusion with enzyme-rich pre-lysosomal vesicles in the late endosome stage, a lysosome forms with degradation of the ingested materials. In the CvME pathway (B), particles that specifically bind to the cell plasma membrane are routed to flask-shaped membrane invaginations that are coated with caveolin. Again, vesicle fission is dynamin-dependent. Rather than processing through endosomes (dashed arrow), the majority of these vesicles form caveosomes, with further processing/routing of the contents based on actions within the endoplasmic reticulum or Golgi apparatus. The NCME pathway(s) (C) continue to be investigated. Cholesterol-rich membrane microdomains or “lipid rafts”, are the specific targets of binding for ligands that stimulate this internalization pathway. Similar to CvME, the lysosomal stage is bypassed with the sparing of the internalized materials from harsh acidic and enzymatic processing. They also appear to be routed to the intracellular membrane organelles.

The uptake and intracellular fate of nanoparticles is highly dependent on the above-mentioned factors but especially to particle size. Larger particles and volumes of the ECF are taken up by phagocytosis and macropinocytosis through two different mechanisms that share a similar intracellular fate (Figure 5). Smaller particles (<150 nm) are taken up and processed via at least three other mechanisms (Figure 6). Cellular uptake mechanisms for liposomes are summarized in Table 1.

Non-phagocytic endocytosis is common to all cells and involves uptake of both fluids and solutes through four main mechanisms: macropinocytosis, clathrin-mediated endocytosis, caveolin-mediated endocytosis, and endocytosis which is independent of clathrin and caveolin [122]. 

Macropinocytosis shares some features with phagocytosis and is a clathrin- and caveolin-independent cellular uptake system that occurs in macrophages as well as many other cell types [129,130,131]. Utilizing membrane protrusions generated by actin interactions in a manner similar to phagocytosis, it differs in the formation of larger endocytic vesicles (typically ranging in diameter from 1–5 µm) through membrane fusion, which nonspecifically samples the ECF and its content. Intracellularly, macropinosomes acidify and shrink, sometimes fusing with lysosomes or recycling their contents to the cell surface.

Clathrin-mediated endocytosis (CME) is essential to cellular homeostasis, allowing uptake of signaling and nutrient macromolecules, and membrane components. Both receptor-mediated and non-specific CME exists, with materials engulfed ending up in degradative lysosomes. CME typically occurs in a membrane region enriched with the cytosolic coat protein clathrin, which polymerizes to form a basket-like framework beneath the cell membrane, causing invagination (clathrin-coated pit, up to 150 nm in depth) and eventual dynamin-mediated formation of a clathrin-coated vacuole (or vesicle) [132,133], with a diameter of 100 nm [134] to 120 nm [130]. The internalization of receptor-ligand complexes via receptor-mediated CME is one of the best defined cellular internalization mechanisms [129], and of paramount importance for various free ligands and nanocarriers bearing targeting ligands (e.g., LDL, transferrin, and epidermal growth factor), and many viruses (e.g., influenza) [122,134,135]. Fluid-phase endocytosis [134], or receptor-independent CME is an internalization pathway for extracellular fluid and its contents that avoids direct binding to the cell membrane components. Another contrasting feature of this pathway is the slower internalization and processing compared to the receptor-mediated CME, with most other features being shared [134]. 

**Table 1:** Cellular uptake mechanisms for liposomes.

Mechanisms of Endocytosis	Primary Cell Types Involved	Opsonization Dependent	Surface Feature-Dependent	Length of Process to Processing	Vesicle Size	Typical Cellular Processing	Other Factors
Phagocytosis	Macrophages Monocytes Neutrophils Dendritic cells	Yes, usually	Yes, increased for both cationic and anionic particles	30 min to 2 h	>250 nm	Acidified, enzyme-rich phagolyso-some.	Increased with hydrophobic, rigid, non-spherical particles. Actin-dependent.
Macropinocytosis	All cells	No	No	-	1–5 μm	Degradative lysosome	Actin-dependent
CME and fluid-phase endocytosis	All cells	No	Enhanced by specific ligands	5–10 min for receptor-mediated. 45–90 min for fluid phase.	<150 nm	Early and late endosomes and eventually degradative lysosomes	Receptor-mediated and non-specific uptake exists. Clathrin- and dynamin-dependent.
CvME	All cells, but especially endothelial cells.	No	Receptor-ligand trafficking on cell surface	20–40 min	<80 nm	Caveosome, avoiding acidic- and enzyme-rich processing.	Caveolin- and dynamin- dependent
CME- and CvME- independent endocytosis	All cells	No	Selected by using targeting ligands specific for “rafts”.	-	<50 nm	Non-lysosomal pathways	Still being investigated.

Caveolin-mediated endocytosis (CvME) features flask-shaped membrane invaginations sized at the lower end of the 50–100 nm range [129,130,134,136], lined by the dimeric protein caveolin. Caveolae (CvME vesicles) are most abundant in endothelial cells, making up 10–20% of the cell surface [130], and allow endocytosis of various proteins and viruses (e.g., SV40), as well as smaller nanocarriers [122]. CvME is more highly regulated than CME, through the involvement of complex signaling on the membrane surface [130,134]. Receptor-ligand interactions on the membrane surface traffic particles to caveolar invaginations [134]. Caveolar fission from the membrane surface is mediated by the GTPase dynamin, a process that is shared with CME [122]. The major differentiation between CME and CvME, besides the typical vesicle size, is the absence of enzymatic activity associated with CvME processing, allowing nanocarriers to by-pass the lysosomal degradation pathways for their payloads (e.g., drugs, peptides, proteins, nucleic acids, *etc.*). The uptake kinetics are not as rapid as seen with CME, but ligands such as albumin, cholesterol, and folic acid are regularly taken up using CvME [134].

While additional clathrin- and caveolin- independent endocytosis pathways have been described, a specific classification for these has only recently been proposed [136]. Like CvME, many, but not all of these pathways involve membrane microdomains, or “rafts”, that are abundant in cholesterol and have diameters of 40–50 nm [130]. Their specific mechanisms and implications in the uptake of nanocarrier systems remain to be better defined.

## 5. Liposomal Toxins

Distinct liposome classes have been developed to package various therapeutic agents for the treatment of cancer, based on structural/pharmacologic features [137]. While most oncologic drugs were initially integrated within the aqueous core of SUVs (see Liposome section), drug incorporation within the liposomal membranes of MLVs further expanded the repertoire of drugs available for liposomal delivery. Drug loading within liposomes is either a passive (drug is incorporated within the vesicle during liposome formation) or an active (addition after vesicle formation) process [138]. Hydrophobic drugs (e.g., taxol and annamycin) can be passively incorporated into liposomes, based on their drug-lipid properties and enhanced by the increased lipid content of MLVs. Drug trapping efficiencies under these conditions can approach 100% for highly lipid soluble agents. In contrast, passive incorporation of hydrophilic drugs (e.g., topotecan, irinotecan) is primarily based on the encapsulated volume of aqueous solution carrying the drug within the vesicle. This internal aqueous volume is increased with vesicle size (e.g., LUV) and typically reduced in MLVs (Figure 3 and Figure 4). Trapping efficiency under these circumstances is typically <30%, due to the liposome size constraints and drug solubility [138], but techniques have been developed to improve hydrophilic drug incorporation results approaching those of lipophilic agents [139]. Active drug incorporation methods into liposomes have been shown to be strongly affected by the drug-buffer composition used as well as the nature of the membrane lipid headgroups [140]. Remote-loading, ion-gradient, intraliposomal stabilization methods of hydrophilic drug incorporation within liposomes have proven effective [141]. Hydrophobic and hydrophilic chemotherapeutic agents can, therefore, be incorporated into liposomes and transported and released over prolonged periods [142,143,144], compared to the non-encapsulated drugs alone. The circulating half-life of these liposomal toxins (LT) can be enhanced further by the addition of a polyethylene glycol (PEG) coat to the liposomal surface, which can also be modified with specific targeting molecules that increase the specificity for receptor-mediated endocytosis, or other cellular incorporation strategies in target cells (see Liposomes, and Cellular Uptake of Liposomes sections above).

## 6. CED of Liposomal Toxins: Pre-clinical Neurooncologic Studies

CED has been used to effectively deliver liposomes and LTs within the CNS in small animals with or without tumors [141,145,146,147,148,149], canines with spontaneous brain tumors [150,151], and nonhuman primates [57,58,152]. From these studies, it has been confirmed that liposomal chemotherapeutics are less toxic and have an extended half-life within brain parenchyma compared to free drugs [153]. Relevant to this discussion, tissue affinities for chemotherapeutic agents delivered via CED were noted to be a limiting factor for parenchymal Vd within the CNS [147], despite being significantly greater than via diffusion-based delivery methods. In this same study [147], drug encapsulation within liposomes significantly increased the effective Vd of the therapeutic. Importantly, alteration of liposomal surface properties (e.g., presence or absence of surface charge, percentage of PEGylation) markedly affected the Vd. Increased liposomal PEGylation yielded the greatest Vd compared to volume of infusion (Vi), probably related to steric stabilization and reduced surface charge [147]. Liposomes delivered via CED within the brain were noted to preferentially traffic in the ECS along white matter tracts, in a path of least resistance, as opposed to passage through more cellular gray matter structures [152,154]. Liposomes were also transported significant distances away from the site of infusion upon gaining entry into the perivascular spaces [154], via a perivascular pump mechanism [48].

Delivery of LTs via CED in rodents harboring brain tumors confirmed higher concentrations of drug at the target site with decreased local toxicity compared to either systemic therapy or CED of non-liposomal drug [146,148]. Efficacy of these methods has been confirmed in rodent tumor models [141,146,148,149], as well as the effective use of mixed liposomes for both drug delivery and contrast agent visualization of the CED process using RCD [57,145,155]. In canines, similar CED-delivered LTs failed to show clinical or histopathological adverse effects in normal [150] or brain tumor-bearing animals [151], while confirming clinical efficacy (Figure 2B–D) and highlighting the importance of RCD to maximize tumor coverage and minimize inappropriate infusions. Convection of gadolinium liposomes (GDL) in nonhuman primate brain has confirmed the lack of toxicity and ability to monitor the infusion process in a larger brain using RCD methods similar to those for humans [58,76]. Recently [156], *in vivo* CED of magnetic nanospheres conjugated to an antibody that selectively binds to the epidermal growth factor receptor (EGFR) mutant (EGFRvIII) found on glioblastoma xenografts, not only allowed specific tumor visualization on MRI, but through an apoptotic mechanism, was associated with targeted cell death with sparing of normal astrocytes. With these and other preclinical data [157], we have argued for the importance of a delivery platform [39] that utilizes RCD to monitor therapeutic distribution, and potential complications associated with CED [62,76] in the neurooncologic patient.

## 7. CED of Liposomal Toxins: Clinical Neurooncologic Studies

Clinical trials featuring LTs in general oncology have been ongoing for over 20 years [158]. With advances in liposomal PEGylation and ligand targeting, improved efficacy and safety for a growing number of LTs (e.g., doxorubicin, acridine, Ara-C, daunomycin, retinoid fenretinide, 5-FdU) has been confirmed [159]. Additionally [160,161,162], novel intraliposomal drug loading and stabilization technologies have allowed incorporation of other therapeutics (e.g., irinotecan, CPT-11), and may lead to additional chemotherapeutic agents being available for clinical development as LTs.

In the brain, use of LTs has been limited over the last decade. PEGylation of some liposomes has been associated with complement activation with repeat injections [163], and the development of complement activation-related psuedoallergy (CARPA) [164,165], which is potentially life-threatening. Initially, liposomes were compared with viral vectors for local direct delivery of genetic payloads to tumors. Although preclinical studies suggested significant transduction rates using liposomal gene therapy vectors, they were generally less efficient than viral vectors [166]. 

The use of liposomes delivered via CED in clinical neurooncology dates back just over 10 years. In an initial phase I/II study [167,168], patients with recurrent glioblastoma multiforme (rGBM) were treated with cationic liposomes containing a suicide gene [169,170], sensitizing tumor cells to systemic ganciclovir therapy. This trial reported no morbidity or mortality associated with the surgical treatment. Liposomal delivery via CED was felt to cause only transient clinical worsening in the patients, possibly related to the infused volume (30 mL over 48 h). Infusion rates varied from 0.025–1.8 mL/h (maximum of 30 µL/min) and were delivered via infusion pump through either one or two implanted silicon catheters within the tumor. Unfortunately, although pre-infusion CED of gadolinium (Gd) contrast attempted to predict the Vd of the therapeutic infusate, it is not clear as to the actual extent of the tumor coverage by the convected therapeutic. The authors actually conclude that the beneficial effect in their patients was restricted to a relatively small volume around the infusion sites [168], making the Vd of the CED suspect.

In another phase I/II trial utilizing a similar cationic liposomal vector/CED protocol for progressive or rGBM, the gene for human interleukin 12 (IL-12) was delivered in an effort to stimulate a local cellular immune antitumor response [171]. Infusion flow rates in this study ranged from 0.1 to 0.5 mL/h (maximum of 8.3 µL/min) until an 11 mL volume was delivered. Although clinical results from this trial were not published, the delivery of similar gene products in neurooncology eventually shifted from initial use of liposomes to the use of viral vectors with this IL-12 paradigm [172], primarily due to the virus’ higher transduction efficiency [98,173].

## 8. Future Directions

Delivery of LTs bearing additional active agents to tumors within the CNS will be forthcoming, based on the significant preclinical experiences to date and the improved methods of incorporation of drugs into liposomes. The further development of MRI contrast-bearing liposomal preparations (e.g., gadoCED) combined with LTs [155], will allow the use of RCD to better document tumor coverage and reduce local complications. 

Recent discussion of retro-convection enhanced delivery (R-CED) techniques [174], and their ability to augment intravenously delivered therapeutics into brain tumors, may promote a systemically administered option for some LTs. A step beyond this concept, yet to be implemented, could include the use of both CED and R-CED to optimally perfuse a local tumor volume and susceptible surrounding brain, using a modification of the push-pull method of cerebral perfusion [175]. 

CED of targeted liposomes carrying a computed tomography (CT) contrast agent for imaging, as well as boron (^10^B) for use in boron neutron capture therapy (BNCT) is progressing [176], and could provide another therapeutic option for human glioma with a real-time imaging option. In this preclinical model [177], transferrin-conjugated PEG liposomes provide selective uptake of ^10^B by the tumor tissue, thereby increasing tumoricidal activity with BNCT. 

Liposomal boron delivery options have been recently reviewed [178], and suggest future therapeutic uses of non-targeted and targeted liposomes in BNCT. Finally, the use of focused low frequency ultrasound (LFUS) to regulate drug release dynamics from LTs [179], suggests a future option for focal parenchymal distribution of liposomally encapsulated therapeutic agents within the Vd provided by CED that may be independent of cell binding/processing. Further investigation of the relative efficacy/toxicity of this approach *versus* a targeted liposomal approach will be required.

## 9. Summary and Conclusions

With the tremendous gains in knowledge regarding liposomal chemistry and cellular processing, the number of therapeutic agents available for delivery within these nanocarriers continues to grow rapidly. Liposomal drug incorporation techniques continue to evolve and provide basic and clinical investigators with more potent and selective LTs for use in oncology. CED provides a precise and effective method for distribution of LTs within the CNS, bypassing the BBB. Together with liposomal contrast agents, LTs convected within a brain tumor and surrounding parenchyma with CED can be directly monitored with MRI, improving the ability to cover the proposed target, avoiding significant leakage from the target site, and providing improved control and safety. 

It remains critical, however, for investigators and clinicians to understand the basics of CED technology prior to considering its use for human trials. A lack of understanding will not allow the proper assessment of this delivery option and prevents a fair comparison to alternatives. Deciphering the mechanisms and critical points associated with CED has been painstakingly worked out over the last 25 years. At a minimum, investigators should have familiarity with how catheter size and shape are essential to minimize tissue trauma and enhance the convection of infusate, while minimizing reflux. Why flow rates are critical to the CED process and should typically not be used above 5 µL/min in an effort to avoid reflux or focal tissue cavitation. The importance of optimal infusion catheter placement within the brain parenchyma, especially related to proximity to ventricular system, subarachnoid space, or tumor resection cavity is critical in maximizing effective Vd. Finally, why the ability to directly visualize the CED process with RCD is essential for reproducible treatment strategies, improved patient safety, and a better determination of therapeutic efficacy, or lack thereof. 

The combination of CED and liposomal technologies is approaching a critical stage in neurooncology, and may finally affect the survival of patients suffering with CNS malignancies (Table 2). In this era of evidence-based medicine, real-time imaging has the opportunity to at least document that the therapeutic agent has been distributed to the target. Incorporation of agents within liposomes for specific intracellular delivery to tumors can reduce the non-specific toxicity of the drug and improve the distribution and contact time within the brain parenchyma. Controlling these parameters will finally allow a clearer picture of comparative drug efficacy, especially if administered via a common platform.

**Table 2:** Essential Components for Combined Use of CED and Liposomes in Neurooncology.

Treatment Modality	Essential Components
CED	Thorough understanding and implementation of parameters to optimize convection.
	Cannula size and shape
	Infusion flow rates
	Specific infusion volumes
	Safe use of contrast agents (free *vs.* liposomal) to visualize the CED process (e.g., RCD).
	Avoid reflux or leakage
	Document Vd, and specific coverage of tumor
Liposomes	Effective use of liposomal technology for improved formulation of LTs.
	Improved understanding of the cellular processing of LTs based on particle size.
	Effective use of lysosomal or non-lysosomal pathways based on delivered therapeutic agent.
	Better appreciation of ultrasound-induced release dynamics and efficacy.
	Effective tumor-specific targeting based on surface ligands.
